# The Protective Effect of Lidocaine on Septic Rats via the Inhibition of High Mobility Group Box 1 Expression and NF-**κ**B Activation

**DOI:** 10.1155/2013/570370

**Published:** 2013-11-25

**Authors:** Huan-Liang Wang, Yan-Qiu Xing, Ying-Xue Xu, Fei Rong, Wei-Fu Lei, Wen-Hua Zhang

**Affiliations:** ^1^Department of Anesthesiology, Qilu Hospital, Shandong University, No. 107 Wenhuaxi Road, Jinan, Shandong 250012, China; ^2^Department of Cardiology, Qilu Hospital, Shandong University, No. 107 Wenhuaxi Road, Jinan, Shandong 250012, China; ^3^Department of Neurosurgery, Qilu Hospital, Shandong University, No. 107 Wenhuaxi Road, Jinan, Shandong 250012, China

## Abstract

Lidocaine, a common local anesthetic drug, has anti-inflammatory effects. It has demonstrated a protective effect in mice from septic peritonitis. However, it is unknown whether lidocaine has effects on high mobility group box 1 (HMGB1), a key mediator of inflammation. In this study, we investigated the effect of lidocaine treatment on serum HMGB1 level and HMGB1 expression in liver, lungs, kidneys, and ileum in septic rats induced by cecal ligation and puncture (CLP). We found that acute organ injury induced by CLP was mitigated by lidocaine treatment and organ function was significantly improved. The data also demonstrated that lidocaine treatment raised the survival of septic rats. Furthermore, lidocaine suppressed the level of serum HMGB1, the expression of HMGB1, and the activation of NF-**κ**B p65 in liver, kidneys, lungs, and ileum. Taken together, these results suggest that lidocaine treatment exerts its protective effection on CLP-induced septic rats. The mechanism was relative to the inhibitory effect of lidocaine on the mRNA expression level of HMGB1 in multiple organs, release of HMGB1 to plasma, and activation of NF-**κ**B.

## 1. Introduction

Sepsis, a systemic inflammatory response syndrome that complicates infection and injury, results in the excessive stimulation on the host immune system and the production of various proinflammatory cytokines. The overproduction of cytokines leads to lethal multiple organ damage [1]. High mobility group box 1 (HMGB1), a nuclear protein widely studied as a transcription factor and growth factor, has recently been identified as a critical mediator of severe sepsis [2, 3]. In addition to its nuclear expression, HMGB1 can be released from inflammatory cells and necrotic tissues during endotoxemia and sepsis [4]. Excessive HMGB1 release has been found to play a key role in the pathogenesis of acute and chronic inflammation. Serum HMGB1 was significantly elevated from 8 to 72 h after endotoxin exposure [2, 3], in comparison with the early mediators, TNF-*α* and IL-1*β*. The treatment with the delayed administration of anti-HMGB1 antibodies [2, 3], a box of HMGB1 [3], and ethyl pyruvate [5] (an inhibitor of HMGB1) beginning as late as the disappearance of plasma TNF-*α* and IL-1*β* significantly increases survival. On the other hand, HMGB1 binds to cell surface receptors once released from the nucleus, and the nuclear factor (NF)-**κ**B signaling pathway may be activated [6]. Thus, the therapeutic window for anti-HMGB1 therapies is significantly wider than that of TNF-*α* targeted interventions, and it may now be possible to develop inhibitors of HMGB1 for the treatment of sepsis [1].

Lidocaine, a common local anesthetic agent, has been known to possess anti-inflammatory effects [7, 8]. It has been shown to modulate inflammatory cascades and provide protection from ischemic reperfusion injury [9–11] and septic peritonitis [12]. It plays a key role in the anti-inflammatory effect on various cell types, including monocytes, macrophages, and neutrophils [7, 8]. The anti-inflammatory effects of lidocaine may be mediated by the inhibition of NF-*κ*B activation and cytokine release [13, 14]. However, the effect of lidocaine on HMGB1 has not been explored *in vitro*.

Cecal ligation and puncture (CLP) model is a well-established rat model of sepsis that resembles the pathophysiology of clinical septic shock. Thus, in the current study, we explored the effect of lidocaine on the treatment of multiple organ injury in CLP-induced septic rats. Furthermore, we ought to determine whether lidocaine could inhibit the expression and release of HMGB1 in CLP-induced septic rats and ultimately explored the possible mechanism underlying this protective effect.

## 2. Materials and Methods

### 2.1. Animals

Adult male Wistar rats weighing 220~250 g were obtained from the Experimental Animals Centre of Shandong University. All experimental protocols were approved by the Experiments Animal Ethics Committee of Shandong University.

The rats were housed four per cage in the regular animal room and given standard laboratory food and tap water *ad libitum. *All rats were anesthetized by intraperitoneal injection of 50 mg/kg sodium pentobarbital and kept under anesthesia during the entire surgical procedures. Three sets of 25 rats were randomly assigned into five groups: the sham-operated group (Sham, *n* = 5), CLP treated with normal saline group (CLP + NS, *n* = 5), CLP treated with lidocaine 3 mg/kg group (CLP + Lido3, *n* = 5), CLP treated with lidocaine 6 mg/kg groups (CLP + Lido6, *n* = 5), and CLP treated with lidocaine 9 mg/kg group (CLP + Lido9, *n* = 5). They were used for PCR, ELISA, and histopathology experiments, respectively (*n* = 5). Another set of 100 rats were assigned into four groups randomly: CLP treated with normal saline group (CLP + NS, *n* = 25), CLP treated with lidocaine 3 mg/kg group (CLP + Lido3, *n* = 25), CLP treated with lidocaine 6 mg/kg groups (CLP + Lido6, *n* = 25), and CLP treated with lidocaine 9 mg/kg group (CLP + Lido9, *n* = 25). They were used for the 7-day survival investigation. Normal saline 0.5 mL or lidocaine (dissolved in normal saline 0.5 mL) was injected intraperitoneally at 10 h after CLP.

### 2.2. Induction of Sepsis

The induction of sepsis was performed using a CLP model as previously described [15]. This procedure reproducibly results in polymicrobial peritonitis, bacteremia, and sepsis. Briefly, under sterile surgical conditions, a 2.0 cm incision was made along the midline of the abdomen to expose the cecum, which was then ligated below the ileocecal junction without causing bowel obstruction. Fecal contents leaked into the peritoneum after the cecum was punctured twice by an 18-gauge needle. To prevent the inadvertent sealing of the puncture, a strip of rubber was inserted into the cecum. Finally, the bowel was returned to the abdomen and the abdominal cavity was closed with a running suture. Sham-operated animals were subjected to laparotomy without cecum ligation and puncture. All rats were immediately given a subcutaneous injection of NS at 50 mL/kg after operation. The rats remained on a heating pad until they awoke.

### 2.3. Organ Tissue Preparation

Rats (five in each group) were killed with sodium pentobarbital 150 mg/kg intraperitoneal at 24 h after CLP. Blood samples were collected before death for serum HMGB1 measurement. Specimens were instantly collected after death. Organs (liver, kidneys, lungs, and ileum) were separately homogenized for ELISA or with TRIzon (50 mg tissue added TRIzon 1 mL) for PCR. For histological examinations and immunohistochemistry, rats were perfused through the left cardiac ventricle with 0.9% NaCl followed by 4% paraformaldehyde at 24 h after CLP. Organs were removed after they became stiff and were placed in a matrix. 2 mm thick tissue slices were taken from each organ and immersed overnight in 4% paraformaldehyde. Each slice was processed into paraffin wax and cut into 5 **μ**m thick sections. The organs from the same rat were used to stain with hematoxylin and eosin for light microscope evaluation and to assay the activation of NF-*κ*B with immunohistochemistry.

### 2.4. Real-Time Polymerase Chain Reaction Analysis (PCR)

Total RNA was extracted from tissues of the liver, kidneys, lungs, and ileum by the TRIzon Reagent kit (TaKaRa Bio Inc., Shiga, Japan). RNA was reverse transcribed using the PrimeScript RT reagent Kit (TaKaRa Bio Inc., Shiga, Japan). Relative expression levels of mRNA were determined using a Roche Diagnostics LightCycler 2.0 Real-Time PCR System (Roche Diagnostics, Shanghai, China) with a SYBR Premix Ex TaqTM (TaKaRa Bio Inc., Shiga, Japan) and gene-specific primers (HMGB1 forward, GGC GAG CAT CCT GGC TTA TC; HMGB1 reverse, AGG CAG CAA TAT CCT TCT CAT AC; GAPDH forward, GGC ACA GTC AAG GCT GAG AAT G; GAPDH  reverse, ATG GTG GTG AAG ACG CCA GTA). A total volume of 10 **μ**L reaction system liquid was subjected to the following PCR program: 1 step of 95°C for 30 s (initial denaturation), followed by 1 step of 41 cycles of 95°C for 5 s, 60°C for 25 s. All protocols were performed according to the manufacturer's instructions.

### 2.5. Immunohistochemistry

After being dried for 45 minutes, paraffin sections were dewaxed in 2 changes of xylene for 15 and 20 minutes each, followed by a descending ethanol series and antigen retrieval in ethylene diamine tetra-acetic acid. The sections were incubated in 3% hydrogen peroxide for 15 min in a humidistat box at room temperature and rinsed in phosphate balanced solution (PBS) for 5 min × 3. After incubating overnight at 4°C with polyclonal rabbit antirat NF-*κ*B p65 (1 : 1000 for liver and lung, 1 : 800 for kidney, and 1 : 1100 for ileum; ab16502; abcam, Cambridge, UK), the sections kept in the humidistat box were warmed to 37°C in an incubator for 45 minutes and then incubated with secondary antibodies (PV-6001, Rabbit two-stage method kit, BIO-LAB, Beijing, China) at 37°C for 30 min. After being washed with PBS for 5 min × 3, the sections were made visible by using 3,3′-diaminobenzidine, terminated in distilled water, and, subsequently, counterstained with hematoxylin for 0.5~1 minute. Then, the slides were differentiated in 1% acid alcohol, blued in 1% ammonia water, dehydrated in graded concentrations of ethanol, cleared in 2 changes of xylene for 10 minutes each, and mounted with neutral gum. The sections were examined and photographed with an Olympus CX21 (Olympus Philippines Inc., Greenhills, Philippines) light microscope at ×400. We evaluated at least four sections from each organ.

### 2.6. Nuclear Localization of NF-*κ*B

The nuclear fraction was collected from liver, lungs, kidneys, and ileum by using the Active Motif nuclear extract kit according to the manufacturer's instructions. NF-*κ*B in the nuclear fraction was measured by using the Active Motif NF-*κ*B family kit according to the manufacturer's instructions.

### 2.7. MPO and DAO Activity Bioassay

The activity of myeloperoxidase (MPO) in lungs and diamine oxidase (DAO) in ileum were determined by using bioassay kits (E90601Ra for MPO; E90656Ra for DAO; Uscn Life Science Inc., Wuhan, China) according to the manuals from the manufacturer.

### 2.8. Measurement of ALT and Creatinine

The contents of serum alanine aminotransferase (ALT) and creatinine were measured by using an automatic biochemistry analyzer (Hitachi7170A, Hitachi High-Technologies Corp., Tokyo, Japan) with commercially available clinical assay kits.

### 2.9. Measurement of Serum HMGB1

Blood samples (0.6 mL) were collected from rats with or without CLP at various time points (0, 2, 4, 8, 16, 24, 36, and 72 h after CLP). The serum was stored at −80°C after centrifuging. The serum HMGB1 levels were determined by ELISA (HMGB1 ELISA Kit II, Shino-Test Corporation, Kanagawa, Japan) according to the manuals from the manufacturer.

### 2.10. Histopathology

Paraffin sections were dewaxed twice in 2 changes of xylene for 15 and 20 min each after being dried for 45 min, followed by a descending ethanol series, and rinsed in running tap water for 2 min. After being stained with hematoxylin for 5 min and washed in tap water, the slides were differentiated in 1% acid alcohol, blued in 1% ammonia water, counterstained with feosin for 1 min, washed in running tap water, dehydrated by a descending ethanol series, cleared in 2 changes of xylene for 10 min each, and mounted with neutral gum. The sections were examined and photographed by an Olympus CX21 light microscope for inflammation and tissue damage.

### 2.11. Survival Studies

Twenty-five rats of each CLP group were enrolled into the survival studies after CLP. All rats had free access to water and food and were frequently observed by research personnel to determine the 7-day survival statistics. The surviving animals were killed with an overdose of diethylether on postoperative day 7. Rats were not given any resuscitation during the experimental protocol.

### 2.12. Statistical Analysis

SigmaPlot version 11.0 (Systat Software, Inc., Chicago, IL) was used for statistical analysis. One-way analysis of variance was used to compare mean values across multiple treatment groups with the Holm-Sidak method. Survival statistical analysis was performed by using a Kaplan-Meier curve and Log-rank (Mantel-Cox) test. A value of *P* < 0.05 was considered significant. There is no significant interexperimental variation determined by two-way *ANOVA.*

## 3. Results

### 3.1. Lidocaine Treatment Protects Organ Injury Induced by CLP

**Figure 1:**
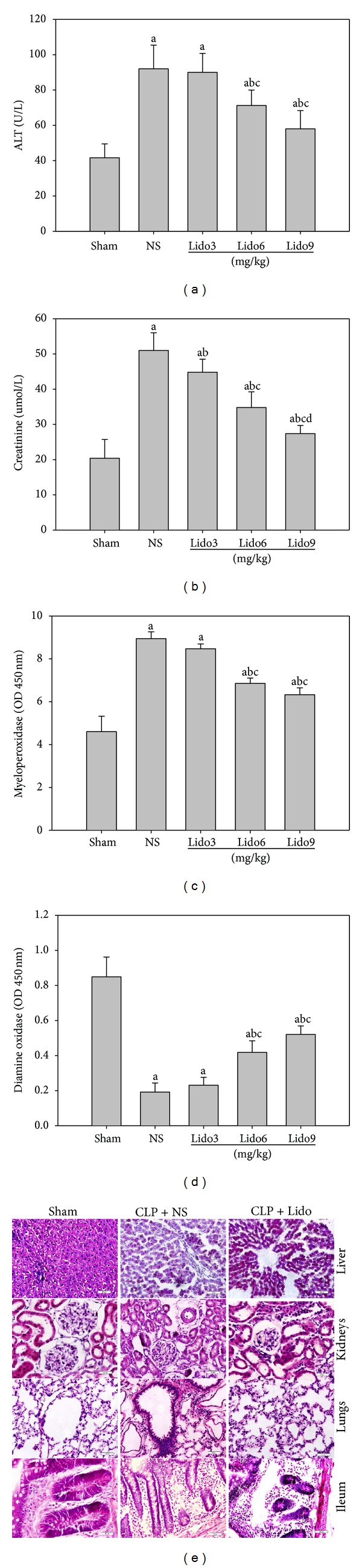
Lidocaine treatment protects acute organ injury induced by CLP. Rats were treated with NS or lidocaine 10 h after CLP. Serum, liver, kidneys, lungs, and ileum were collected 24 h after CLP. Plasma contents of ALT (a) and creatinine (b), activity of MPO in lungs (c), and DAO in ileum (d) were measured. Data were presented as mean ± SD (*n* = 5). ^a^ *P* < 0.05 versus Sham; ^b^ *P* < 0.05 versus NS; ^c^ *P* < 0.05 versus Lido3 mg/kg; ^d^ *P* < 0.05 versus Lido6 mg/kg. (e) Hematoxylin and eosin stained sections of liver, kidneys, lungs, and ileum from rats subjected to Sham and to CLP treated with NS and rats subjected to CLP treated with lidocaine 9 mg/kg at 24 h after CLP. Original magnification: ×400. NS: normal saline; Lido: lidocaine.

To determine the effect of lidocaine on CLP-induced organ injury, lidocaine was intraperitoneally administrated to rats 10 h after CLP. Serum ALT and creatinine levels, lung MPO activity, and ileum DAO activity were determined 24 h after CLP. As shown in Figures 1(a), 1(b), and 1(c), the serum levels of ALT and creatinine and the activity of lung MPO significantly increased in all groups subjected to CLP compared to the sham group (*P* < 0.05). However, rats treated with lidocaine at 6 and 9 mg/kg had significantly lower levels of plasma ALT (*P* < 0.05), creatinine (*P* < 0.001), and lung MPO activity (*P* < 0.001) compared to rats treated with NS, and lidocaine at 3 mg/kg also produced a significant decrease (*P* < 0.033) in plasma creatinine. Also, the activity of ileum DAO significantly decreased in all groups subjected to CLP compared to the sham-operated group (*P* < 0.05). Rats treated with lidocaine at 6 and 9 mg/kg had a significantly higher level of ileum DAO activity compared to rats treated with NS (*P* < 0.001, Figure 1(d)). Next, histopathological examinations of liver, kidneys, lungs, and ileum were used to determine the effect of lidocaine on CLP-induced organ injury. As shown in Figure 1(e), these morphological and acute inflammatory changes were attenuated in the group treated with lidocaine at 9 mg/kg compared to the group treated with NS. The major acute inflammatory injuries in the liver from CLP-induced septic rats were extensive hepatic tissue malformation, intracellular and interstitial edema, and large area of necrosis. Those in the kidneys included interstitial inflammatory cell infiltration, kidney tubular hyperemia, endothelial cell swelling, and intercapillary cell proliferation. The major morphological alterations in CLP-induced lungs included the infiltration of leukocytes and leakage of erythrocytes into alveolar and interstitial spaces, edema, alveolar distortion, and thickening of the alveolar-capillary membrane. The alterations in ileum included villi edema, considerable shortening, not uniform in length and arranged in a disorderly manner, epithelium flaking off, and lamina propria edema accompanied by inflammatory cell infiltration. In contrast, rats treated with lidocaine showed minor liver, lungs, kidneys, and ileum damage. Altogether, lidocaine treatment protects acute organ injury induced by CLP.

### 3.2. Lidocaine Treatment Improves the Survival of CLP-Induced Septic Rats

**Figure 2:**
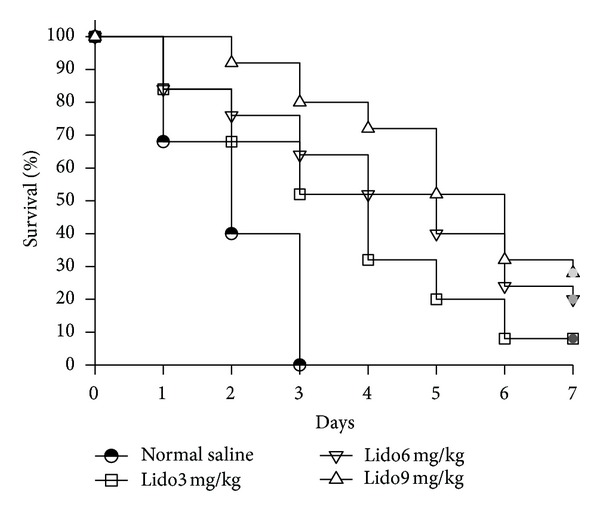
Lidocaine treatment improves the survival of CLP-induced septic rats. The seven-day survival rates were performed on rats treated with NS or lidocaine. Log-rank analysis demonstrated a significant improvement in survival for lidocaine 3, 6, and 9 mg/kg versus NS (*χ* ^2^ = 13.309; *χ* ^2^ = 19.145; *χ* ^2^ = 34.786, *P* < 0.001). NS: normal saline; Lido: lidocaine.

To explore the effect of lidocaine on the survival of CLP-induced septic rats, the 7-day survival rates were performed to determine whether lidocaine treatment protected rats from CLP-induced sepsis. The results demonstrated a significant prolongation of survival in rats treated by 3, 6, and 9 mg/kg of lidocaine (*P* < 0.001) as shown in Figure 2.

### 3.3. Lidocaine Treatment Prevents Systemic HMGB1 Release in CLP-Induced Septic Rats

**Figure 3:**
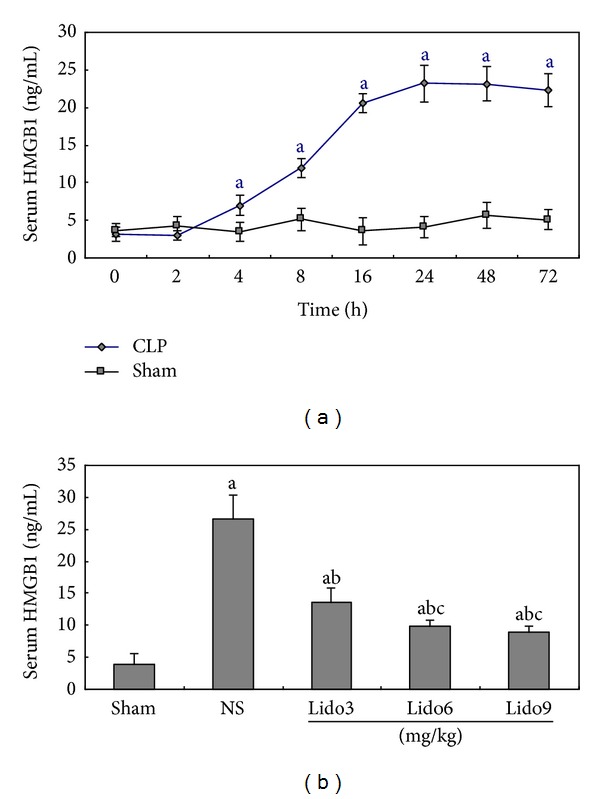
Lidocaine treatment decreases of systemic HMGB1 release in CLP-induced septic rats. (a) Time course of changes in serum HMGB1 levels. Serum from rats with or without CLP was collected at various time points (0, 2, 4, 8, 16, 24, 48, and 72 h after CLP). Serum HMGB1 levels were measured by ELISA. Data were presented as mean ± SD (*n* = 5). ^a^ *P* < 0.05 versus Sham. (b) Serum from rats subjected to CLP treated with NS and rats subjected to CLP treated with lidocaine 3, 6, and 9 mg/kg at 24 h after CLP. Serum HMGB1 levels were measured by ELISA. Data were presented as mean ± SD (*n* = 5). ^b^ *P* < 0.05 versus NS; ^c^ *P* < 0.05 versus Lido3 mg/kg. NS: normal saline; Lido: lidocaine.

To investigate the effect of lidocaine on systemic HMGB1 release in CLP-induced septic rats, serum samples were collected for the determination of HMGB1 levels by ELISA. Serum levels of HMGB1 were elevated at 4 h and reached the peak value at 12–16 h after CLP as shown in Figure 3(a). Rats receiving lidocaine treatment exhibited significant reduced serum HMGB1 level as compared with the control group (Figure 3(b)). This indicates that lidocaine treatment inhibited HMGB1 release to the plasma.

### 3.4. Lidocaine Treatment Inhibits the Expression of HMGB1 in Multiple Organs of CLP-Induced Septic Rats

**Figure 4:**
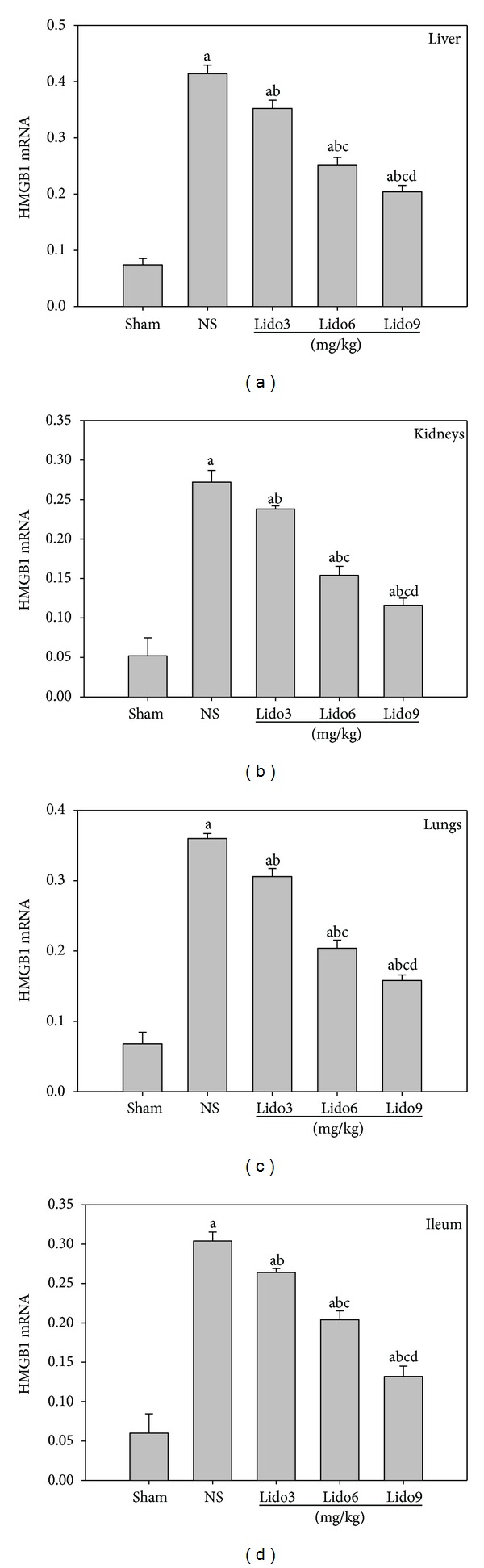
Lidocaine treatment inhibits HMGB1 mRNA expression in multiple organs of CLP-induced septic rats. Rats were treated with NS or lidocaine 10 h after CLP. Liver, kidneys, lungs, and ileum were collected 24 h after CLP. HMGB1 mRNA expression in various organs were measured by Real-Time PCR. Data were presented as mean ± SD (*n* = 5). ^a^ *P* < 0.001 versus Sham; ^b^ *P* < 0.001 versus NS; ^c^ *P* < 0.001 versus Lido3 mg/kg; ^d^ *P* < 0.001 versus Lido6 mg/kg; NS: normal saline; Lido: lidocaine.

To further explore the possible mechanism by which lidocaine decreases serum HMGB1 level and attenuates organ injury induced by CLP, we next tested the effect of lidocaine on HMGB1 transcription. Thus, the total RNA was exacted from liver, lungs, kidneys, and ileum after CLP, and the HMGB1 mRNA level was determined by real-time quantitative PCR. Data indicated that the level of HMGB1 mRNA in the organs of rats undergoing CLP was significantly increased in comparison with the sham group and decreased when treated with lidocaine in a dose-dependent manner at 24 h after CLP as shown in Figure 4.

### 3.5. Lidocaine Treatment Inhibits Translocation and Activity of NF-*κ*B in Multiple Organs of CLP-Induced Septic Rats

**Figure 5:**
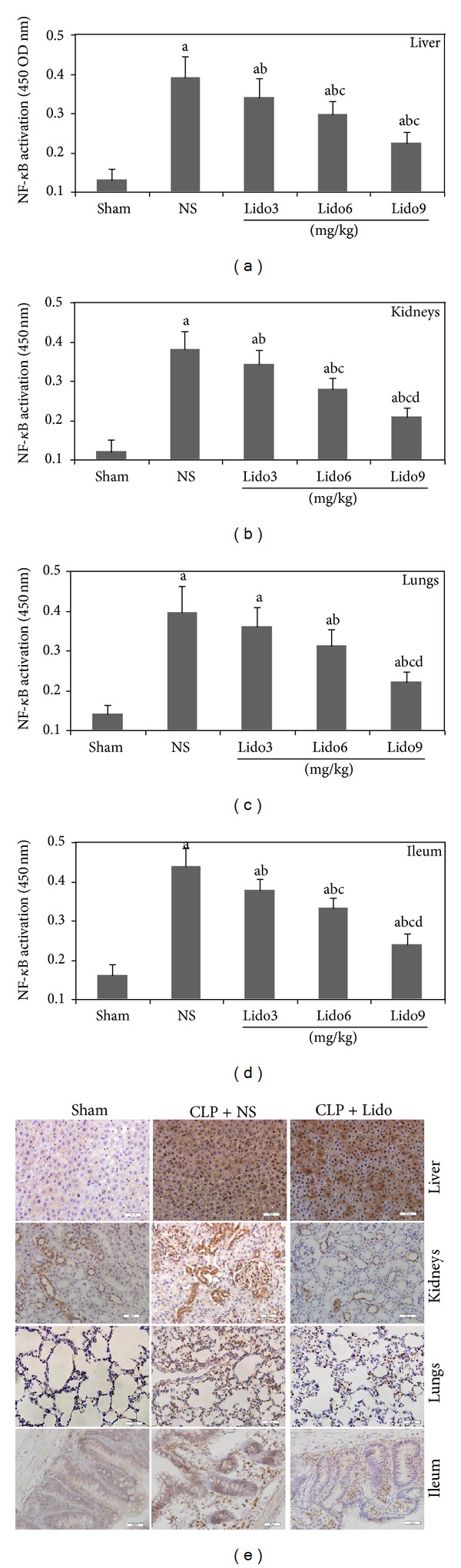
Lidocaine treatment inhibits translocation and activity of NF-*κ*B in organs of CLP-induced septic rats. Rats were treated with NS or lidocaine 10 h after CLP. Liver, kidneys, lungs, and ileum were collected 24 h after CLP (a, b, c, and d). The nuclear fraction was collected and NF-*κ*B levels in the nuclear fraction were measured using the Active Motif NF-*κ*B family kit. Data are presented as mean ± SD (*n* = 5). ^a^ *P* < 0.001 versus Sham; ^b^ *P* < 0.001 versus NS; ^c^ *P* < 0.001 versus Lido3 mg/kg; ^d^ *P* < 0.001 versus Lido6 mg/kg. (e) Immunohistochemical staining of nuclear factor-**κ**B p65 in liver, kidneys, lungs, and ileum from rats subjected to Sham and to CLP treated with NS and rats subjected to CLP treated with lidocaine 9 mg/kg at 24 h after CLP. Original magnification: ×400; NS: normal saline; Lido: lidocaine.

The above results demonstrated that lidocaine inhibited HMGB1 mRNA expression in multiple organs of septic rats, so we next investigate whether lidocaine affect the upstream signal transduction pathway of NF-*κ*B as well. The NF-*κ*B DNA binding activity was assessed using the ELISA-based kit. As shown in Figures 5(a), 5(b), 5(c), and 5(d), lidocaine treatment significantly inhibited the NF-*κ*B translocation to nucleus induced by CLP in liver, kidneys, lungs, and ileum. Furthermore, immunohistochemistry was used to determine the nuclear fraction NF-*κ*B activity. The immunohistochemical staining of NF-*κ*B p65 of the sham group was mainly in the cytoplasm with light brown coloration in the liver, kidneys, lungs, and ileum (Figure 5(e)). However, the staining of NF-*κ*B p65 of rats subjected to CLP treated with NS was dark brown and the nucleus could not be clearly seen because of the strong staining of NF-*κ*B p65. But it became lighter and the nucleus could be clearly seen in blue in the group treated with lidocaine 9 mg/kg. Taken together, the aforementioned data suggest that the Lidocaine treatment inhibits translocation and activity of NF-*κ*B in multiple organs of CLP-induced septic rats.

## 4. Discussion

Although sepsis is the leading cause of hospitalized death, the therapeutic choices are limited. The mortality in sepsis is strongly influenced by the development of organ injury and dysfunction. Death from severe sepsis occurs because organs become dysfunctional, especially the lungs, liver, and kidneys [16]. Gallos et al. [12] found that chronic local anesthetic infusion reduced the magnitude of CLP-induced renal and hepatic injury. In this study, the functions of various organs after CLP were evaluated. Serum ALT, creatinine, and lungs MPO activity elevations which reflect liver, kidneys, and lungs dysfunction, respectively, were significantly reduced in the lidocaine treatment group. DAO activity reflecting ileum function significantly increased in the lidocaine treatment group. Combining these results with histopathology demonstrates that lidocaine treatment can significantly improve the function not only of the liver and kidneys but also of the lungs and small intestine in septic rats induced by CLP. At the same time, the results of survival rate demonstrate that lidocaine treatment significantly improves the survival rate in CLP-induced septic rats. Thus, lidocaine has a protective effect on multiple organ injury in sepsis induced by CLP.

HMGB1 has previously been reported to be a cytokine that mediates organ damage in severe sepsis [2, 3]. The administration of HMGB1 to experimental animals causes lethal organ damage via a mechanism that is dependent on the development of epithelial cell dysfunction, activation of macrophages/monocytes to release proinflammatory cytokines, upregulation of endothelial adhesion molecules, stimulation of epithelial cell barrier failure, and mediation of fever and anorexia [2, 17]. Previous studies demonstrated that a positive correlation was observed between HMGB1 and multiple organ system failure score in patients with septic shock [18]. Furthermore, serum ALT [18, 19], creatinine [19], and lung MPO activity [18, 19] manifested a significant positive correlation with HMGB1 mRNA in the liver, kidneys, and lungs, whereas there was no correlation between tissue TNF-*α* mRNA expression and organ damage [18]. Data from our present study suggests that an increased HMGB1 mRNA expression in the liver, kidneys, lungs, and ileum is associated with the damage of acute organ injury as shown by significantly elevated levels of plasma ALT, creatinine and activity of MPO in lungs, decreased activity of DAO in the ileum mucous membrane, and inflammatory histological alteration secondary to sepsis induced by CLP. Furthermore, lidocaine treatment dramatically relieves mRNA expression in various organs and the level of serum HMGB1 in CLP-induced septic rats. Therefore, the protective effect of lidocaine on septic acute organ injury might at least partly result from its attenuating mRNA expression of tissue HMGB1 and the release of systemic HMGB1.

The precise mechanism of anti-inflammatory effects of lidocaine has not been fully elucidated. NF-*κ*B is a multisubunit molecular that belongs to the Rel family of transcription and its activation is triggered by proinflammatory cytokine stimulation of endothelial cells [20]. Previous studies indicated that the downregulation of NF-*κ*B resulted in the decreased expression of many proinflammatory cytokines [13, 14]. Therefore, NF-*κ*B activation plays an important role in the inflammatory response during sepsis [21, 22]. Lee et al. [23] demonstrated that the blocking of voltage-sensitive sodium channels and inhibition of p38 mitogen-activated protein kinase might be involved in the inhibitory effect of lidocaine on the NF-*κ*B signaling pathway in RAW264.7 cells. However, Lang et al. [20] reported that lidocaine could inhibit IL-8 and IL-10 secretion from intestinal cells via the inhibition of NF-*κ*B activation and decreased IkappaB phosphorylation. Our data suggest that lidocaine inhibits both the NF-*κ*B signal pathway and in the tissue mRNA levels of HMGB1 and the release of systemic HMGB1. Given the complexity of NF-*κ*B activation, further study will be required to explore the upstream transduction pathway leading to the inhibitory NF-*κ*B by lidocaine. Recent thinking surrounding HMGB1 stated that HMGB1 by itself has relatively little biological activity. HMGB1 may form complexes with other signaling molecules such as single-stranded oligonucleotides and IL-1*β* to form highly inflammatory complexes which will trigger or sustain inflammation [24–26]. Thus, we speculate that the effect of lidocaine on HMGB1 may result in the reduction of complexes of HMGB1/other signaling molecules.

In summary, our current data demonstrate that lidocaine exerts its protective effection on CLP-induced septic rats. The mechanism is relative to the inhibitory effect of lidocaine on the mRNA expression level of HMGB1 and release of HMGB1 to plasma and activation of NF-*κ*B. These findings will facilitate further investigation in the therapeutic approach of sepsis and other serious inflammatory diseases.
